# Clinical Relevance of Peripheral Interleukins in Drug-Naive First-Episode Psychosis: Symptom-Specific Associations from the PANSS Dimensions

**DOI:** 10.3390/brainsci15090932

**Published:** 2025-08-27

**Authors:** Iva Binic, Jovana Petrovic, Olivera Zikic, Suzana Tosic Golubovic, Vladimir Djordjevic, Marko Stevanovic, Dane Krtinic, Marija Andjelkovic Apostolovic

**Affiliations:** 1 Department of Psychiatry, Medical Faculty, University of Nis, 18000 Niš, Serbia; jovanapetrovic0912@gmail.com (J.P.); okac55@gmail.com (O.Z.); suzanatosic67@gmail.com (S.T.G.); vlajce74.vdj@gmail.com (V.D.); 2 Psychiatry Clinic, University Clinical Center Nis, 18000 Niš, Serbia; 3 Center for Mental Health Protection, University Clinical Center Nis, 18000 Niš, Serbia; 4 Special Hospital for Psychiatry Diseases “Gornja Toponica”, 18202 Niš, Serbia; markostevanovic220@yahoo.com; 5 Department of Pharmacology and Toxicology, Faculty of Medicine, University of Nis, 18000 Niš , Serbia; kdane86@gmail.com; 6 Oncology Clinic, University Clinical Center Nis, 18000 Niš, Serbia; 7 Department of Medical Statistics and Informatics, Faculty of Medicine, University of Nis, 18000 Niš, Serbia; drmari84@gmail.com; 8 Institute for Public Health, 18000 Niš, Serbia

**Keywords:** first episode psychosis, drug-naive, cytokine, interleukin, PANSS

## Abstract

**Background/Objectives**: Emerging evidence suggests a role of immune–inflammatory mechanisms in the pathophysiology of schizophrenia, particularly in the early stages of the illness. Cytokines, as key mediators of inflammation, may affect brain function and clinical presentation. Drug-naive patients with first-episode psychosis (FEDN) offer a unique opportunity to investigate these associations free from confounding pharmacological effects. **Methods**: This study included 38 patients with drug-naive first episode psychosis and 22 age- and sex-matched healthy controls. Serum concentrations of IL-1β, IL-2, IL-6, and IL-10 were measured using ELISA. Clinical symptoms were assessed using the PANSS scale. Statistical analyses included Mann–Whitney U tests, Spearman’s correlations, and ROC curve analysis. **Results**: Significantly elevated serum levels of IL-1β, IL-2, and IL-10 were observed in the FEDN group compared to the controls (*p* < 0.01), while IL-6 levels did not differ significantly. IL-2 exhibited the highest discriminatory power in differentiating the patients from the controls (AUC = 0.917; 95% CI: 0.759–1000.0; *p* < 0.001). IL-1β levels positively correlated with negative and general psychopathology symptoms, including hostility and grandiosity. IL-10 was associated with volitional disturbance and overall PANSS severity. **Conclusions**: Our findings underscore the relevance of immune dysregulation in the early stages of psychosis and highlight the potential of specific cytokines, particularly IL-2 and IL-1β, as peripheral biomarkers. Their diagnostic utility and correlation with symptom dimensions suggest a promising role in the development of precision psychiatry approaches, including early detection strategies and individualised therapeutic targeting. Longitudinal studies are needed to validate these findings and to assess their prognostic significance.

## 1. Introduction

Schizophrenia and other psychotic disorders are considered complex, heterogeneous conditions whose aetiology involves a multifaceted interaction of genetic predispositions, adverse environmental factors, and disturbances in the development of the nervous system [1,2,3]. Traditional explanatory models, which have dominated for decades, have emphasised dopaminergic dysfunction as the central mechanism, while more recent findings in the field of pathophysiology have increasingly highlighted glutamatergic dysfunction, oxidative stress, neurotoxicity, and, more frequently, immune–inflammatory processes [4].

A significant body of research over the past two decades has indicated that patients with psychosis—especially in the early stages of the illness—are prone to inflammatory changes in both the central and peripheral nervous systems [5]. The hypothesis of an inflammatory contribution to the etiopathogenesis of psychosis suggests that immune system activation—whether through genetic predispositions [6], exposure to prenatal and perinatal stressors [7], infections, or psychosocial stress [8,9]—can influence the development and course of the illness.

Cytokines, as key mediators of inflammation, can act as neuromodulators, cross the blood–brain barrier, and affect neurotransmitter systems (dopaminergic, glutamatergic, serotonergic) and neuroplasticity, as well as the function of microglia and astrocytes [10,11,12,13].

The first psychotic episode (FEP) represents a critical window for investigating these mechanisms, as the clinical presentation at this stage has not yet been altered by long-term pharmacotherapy or chronic neurodegenerative processes. A particularly important subgroup consists of patients who have not received antipsychotic treatment up to that point (“drug-naive”), as biomarkers identified in them are more likely to reflect the underlying pathophysiological mechanisms, rather than the effects of treatment or chronic dysfunction [14].

In this context, examining the interleukins (IL-1β, IL-2, IL-6, IL-10) in these patients offers a promising path toward understanding the early immunological disturbances associated with psychosis. Previous meta-analyses and original studies have shown that the levels of certain pro-inflammatory cytokines (e.g., IL-1β, IL-2, IL-6, TNF-α) are significantly higher in patients with schizophrenia compared to healthy individuals, while the levels of anti-inflammatory cytokines, such as IL-10, are often decreased—or, conversely, elevated—as a reflection of compensatory mechanisms [15,16,17,18,19,20]. However, the data are often heterogeneous, which may be due to varying diagnostic criteria, stages of the disease, presence of comorbidities, medication use, and differing methodological approaches.

An increasing number of studies are attempting to clarify whether inflammatory status has a specific clinical reflection—that is, whether the levels of individual cytokines are associated with specific psychotic symptoms. In this light, investigating the specific relationship between peripheral interleukin levels and the clinical presentation in patients with FEDN psychosis may contribute to a better understanding of the disease’s pathophysiology, as well as to the development of a personalised treatment approach. The clinical relevance of such biomarkers lies in their potential predictive and diagnostic value, as well as in the possibility of developing immunomodulatory therapeutic strategies in the future [20,21].

This study aims to examine whether there was a difference in the concentrations of IL-1β, IL-2, IL-6, and IL-10 between the patients experiencing a first episode of psychosis who have not previously been treated with antipsychotics and the healthy controls, as well as to determine the potential association of these biomarkers with the intensity and structure of the clinical presentation as assessed by the PANSS scale. These interleukins were selected due to their established roles in the innate immune response and their previously reported involvement in psychosis-related immune dysregulation.

## 2. Materials and Methods

This prospective observational study was conducted at the Clinic of Psychiatry of the University Clinical Centre Niš from 2023 to 2025. It was carried out following the principles of Good Clinical Practice and the Declaration of Helsinki and was approved by the Ethics Committee of the University Clinical Centre Niš, the Ethics Committee of the Faculty of Medicine at the University of Niš, and the Ethics Committee of the Institute for Transfusion Niš.

### 2.1. Participants

This study included a total of 60 participants, of whom 38 were patients with a drug-naive first-episode (FEDN) psychosis, diagnosed according to the ICD-10 criteria (F23.1 and F23.2), and 22 were healthy volunteers who donated blood at the Institute for Transfusion Niš. All patients were hospitalised under full-time treatment and had not previously been exposed to psychopharmacological therapy. The control group had no history of psychiatric disorders or chronic somatic diseases and was not receiving any treatment that could affect the inflammatory parameters.

The inclusion criteria for the patient group were as follows: age, between 18 and 50 years; both sexes; first episode of psychosis from the schizophrenia spectrum; absence of prior pharmacological intervention; presence of symptoms requiring hospitalisation; and the ability to provide informed consent. Healthy participants in the control group had to be within the same age range, without any acute or chronic medical or psychiatric conditions.

The exclusion criteria for all participants included the following: the presence of autoimmune, autoinflammatory, immunodeficiency, malignant, or metabolic diseases; current or recent use of corticosteroids, immunomodulatory, or anti-inflammatory therapy; active infection at the time of sampling; significant obesity (BMI > 30); as well as regular consumption of alcohol, tobacco, or psychoactive substances. Special attention was given to eliminating factors that could affect systemic inflammation independently of the psychotic disorder.

### 2.2. Blood Sampling and Laboratory Procedures

Basic demographic data, body weight, height, and body mass index (BMI) were recorded for all participants. Blood samples were collected in the morning, before the initiation of any therapy in the patients, via venipuncture of the antecubital vein into EDTA-containing tubes. After centrifugation, the samples were stored at −80 °C until the time of analysis. The concentrations of IL-1β, IL-2, IL-6, and IL-10 were determined using commercial ELISA kits produced by Wuhan Fine Biotech, following the manufacturer’s protocol. Calibration ranges and limits of detection (LOD) were as follows: IL-1β: 3.906–250 pg/mL (LOD 2.344 pg/mL); IL-2: 15.625–1000 pg/mL (LOD 9.375 pg/mL); IL-6: 4.688–300 pg/mL (LOD 2.813 pg/mL); IL-10: 7.813–500 pg/mL (LOD 4.688 pg/mL). All results were within the calibrated range, no dilution was required, and all samples were run in duplicate with CVs within the manufacturer’s specifications.

### 2.3. Clinical Assessment

The psychiatric evaluation was conducted on the same day as the blood sampling and included the standardised diagnostic interview MINI (Mini International Neuropsychiatric Interview), which confirmed psychotic disorders and excluded comorbid diagnoses. To assess the severity of the clinical presentation, the PANSS scale was used, which allows for a quantitative evaluation of positive, negative, and general symptoms of psychosis.

### 2.4. Statistical Analysis

A statistical analysis was performed using the SPSS software package, version 22.0. Descriptive statistical parameters were used for numerical data. Normality of data distribution was assessed using the Shapiro–Wilk test. All four interleukins (IL-1β, IL-2, IL-6, and IL-10) deviated from normal distribution, and appropriate non-parametric tests (Mann–Whitney U) were used for between-group comparisons. Differences between the groups were assessed using the *t*-test, Mann–Whitney U test, ANOVA, or Kruskal–Wallis test, depending on the normality of the data distribution. Correlation analyses were conducted using Spearman’s and Pearson’s tests, and ROC analysis was used to evaluate the diagnostic value of the interleukins. The strength of all statistically significant correlations between interleukin levels and clinical symptom severity scores has been classified according to Chaddock’s scale: negligible (<0.10), weak (0.10–0.29), moderate (0.30–0.49), strong (0.50–0.69), and very strong (≥0.70). Statistical significance was set at the level of *p* < 0.05 (two-tailed). Sample size was determined prior to data collection using G*Power software (version 3.1.9.2), based on previously published data on cytokine levels. With a power of 95% and a significance level of 0.05, the required minimum sample was calculated to be 32 participants. This corresponds to a beta error probability of 0.05, indicating a low risk of Type II error for detecting significant between-group differences in cytokine levels.

## 3. Results

### 3.1. Sociodemographic Characteristics

This study included 60 participants—38 patients with a drug-naive first-episode psychosis and 22 healthy volunteers. The groups were comparable in terms of sex and age, with no statistically significant differences (*p* > 0.05). There were also no differences in place of residence and education level. Significant differences were observed in marital status and parenthood: patients were more often unmarried and childless compared to the healthy controls (*p* < 0.01). The most pronounced difference was related to employment, with the majority of patients being unemployed (*p* < 0.001). The details are presented in Table 1.

### 3.2. Serum Concentrations of Interleukins in Patients and Healthy Controls

**Figure 1:**
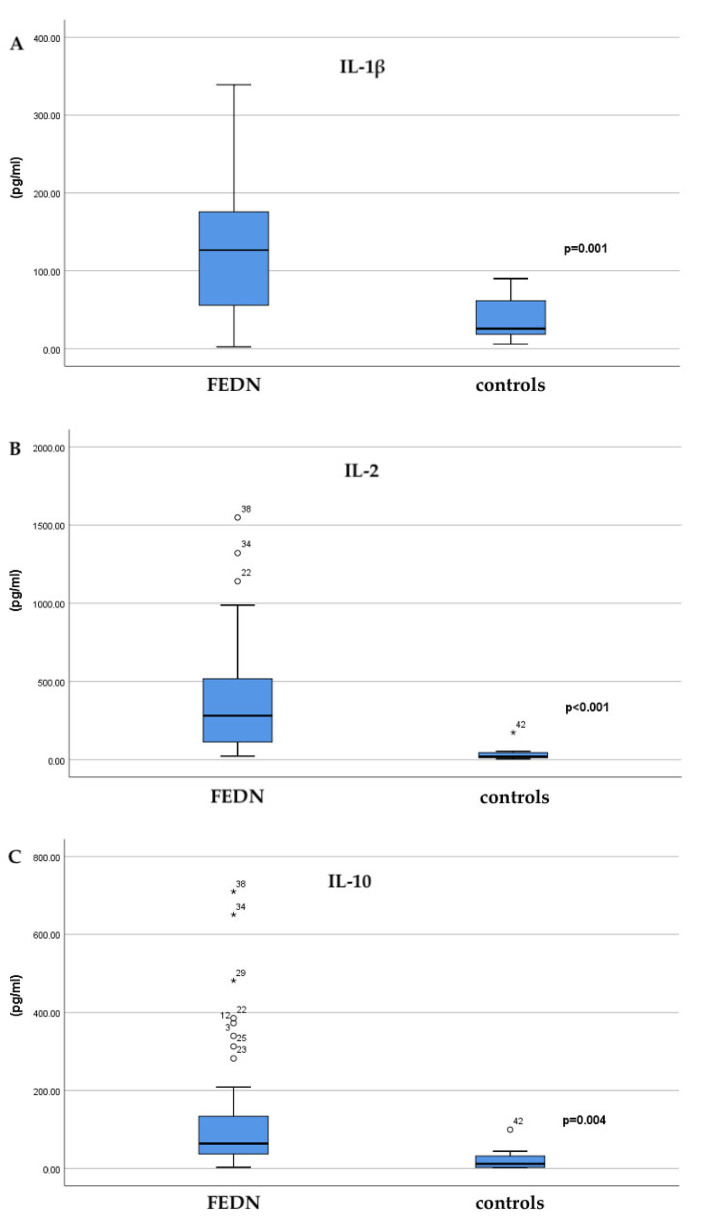
Serum concentrations of interleukins IL-1β (**A**), IL-2 (**B**), and IL-10 (**C**) in the patients with drug-naive first-episode psychosis (FEDN) and the healthy controls. (**A**) IL-1β concentrations were significantly higher in the patient group compared to the controls (*p* = 0.001). (**B**) IL-2 levels also showed a marked increase in the patients, with minimal overlap between the groups (*p* < 0.001). (**C**) IL-10, an anti-inflammatory cytokine, was significantly elevated in the patient group, with a wide range of values reflecting immune response heterogeneity (*p* = 0.004). Data are presented as medians with interquartile ranges (25th–75th percentiles). Interleukin-6 was not included in this figure because no significant between-group difference was observed (see Table 2).

Within our study, statistically significant differences were observed between the patients with FEDN psychosis and the healthy controls for IL-1β, IL-2, and IL-10, whereas the IL-6 levels did not differ significantly (*p* = 0.698). (Table 2). The analysis of interleukin concentrations showed significant differences between the patients with a drug-naive first-episode psychosis and the healthy controls, suggesting the presence of pronounced immunological changes in the patients. The findings for IL-1β stood out in particular, with significantly higher levels observed in the patient group (130.14 ± 93.22 pg/mL) compared to the healthy controls (37.06 ± 27.61 pg/mL; *p* = 0.001). In Figure 1A, which shows the distribution of the IL-1β values, it is visible that the patients almost consistently have elevated concentrations compared to the healthy subjects, further confirming the statistical significance of the findings. Similarly, IL-2 was also significantly elevated in the patients (395.7 ± 380.29 pg/mL) compared to the controls (42.15 ± 55.59 pg/mL; *p* < 0.001). Figure 1B, which displays the IL-2 values, further illustrates this difference, with a clear separation of patient concentrations from those recorded in the healthy population. In addition to the pro-inflammatory cytokines, IL-10, which has an anti-inflammatory effect, was also significantly higher in the patient group (140.65 ± 176.84 pg/mL) than in the control group (24.20 ± 33.43 pg/mL; *p* = 0.004). Figure 1C, which displays the IL-10 values, indicates a wide range of concentrations in the patient group, reflecting the heterogeneity of the immune response during a psychotic episode, while also confirming a statistically significant difference compared to the healthy control group. In contrast to the previously mentioned cytokines, IL-6 did not show a statistically significant difference in concentrations between the groups (9.13 ± 12.90 pg/mL in patients vs. 5.01 ± 4.28 pg/mL in the controls; *p* = 0.698). Although the mean values were higher in the patient group, the variability and overlap with values from the control group reduced the statistical strength of this finding.

### 3.3. Diagnostic Value of Interleukins—ROC Analysis

To investigate the potential of peripheral interleukins as biomarkers for distinguishing patients with a first-episode psychosis from the healthy controls, an ROC analysis was performed for each cytokine. The results showed that IL-1β had significant discriminative value, with an area under the curve (AUC) of 0.842 (95% CI: 0.659–0.981; *p* < 0.001), indicating a good ability to differentiate between the studied groups. IL-2 demonstrated an even higher diagnostic value, with an AUC of 0.917 (95% CI: 0.759–1000.0; *p* < 0.001). IL-10 also showed a statistically significant discriminative ability (AUC = 0.817; 95% CI: 0.565–1000.0; *p* < 0.001). By contrast, IL-6 did not show significant discriminative ability (AUC = 0.396; 95% CI: 0.187–0.635; *p* = 0.451) (Table 3).

### 3.4. Association of Interleukins with the PANSS Scale Scores

The analysis of the association between serum interleukin concentrations and the PANSS scale scores revealed several statistically significant findings, as presented in Table 4. IL-1β showed a positive correlation with the negative subscale (ρ = 0.374, *p* = 0.021), a positive correlation with the general psychopathology scale (ρ = 0.339, *p* = 0.037), a significant negative correlation with the items “grandiosity” (ρ = −0.418, *p* = 0.009) and “hostility” (ρ = −0.343, *p* = 0.033) from the positive subscale. IL-2 showed no significant correlation with the total PANSS score (ρ = 0.199, *p* = 0.233) or with the individual subscales; however, it had a significant positive relationship with the item “unusual thought content” (ρ = 0.360, *p* = 0.026). IL-6 demonstrated a positive correlation with the item “excitement” (ρ = 0.350, *p* = 0.030), but no statistically significant associations with the total PANSS score or its subscales. IL-10 showed a positive correlation with the total PANSS score (ρ = 0.382, *p* = 0.017), a positive correlation with the general psychopathology scale (ρ = 0.390, *p* = 0.015), and a positive correlation with the item “disturbance of volition” (ρ = 0.354, *p* = 0.029). IL-1β was moderately correlated with negative symptoms (ρ = 0.374) and general psychopathology (ρ = 0.339) according to Chaddock’s scale. IL-10 showed a moderate positive correlation with total PANSS score (ρ = 0.382) and disturbance of volition (ρ = 0.354).

## 4. Discussion

The focus of this study was to examine the clinical significance of peripheral interleukins (IL-1β, IL-2, IL-6, and IL-10) in previously untreated patients with FEDN psychosis, by comparing them with the healthy controls and by analysing their association with symptoms assessed using the PANSS scale. Unlike previous studies that included heterogeneous samples of patients with varying illness durations and prior antipsychotic treatments, our sample consists exclusively of drug-naive patients who had not received any prior pharmacotherapy. This approach allows for a more reliable exploration of the potential role of inflammatory cytokines in the early stages of psychosis, without the confounding effects of medication. Additionally, strict inclusion and exclusion criteria were applied to minimise the impact of factors that could modulate the immune response. All patients were non-smokers, had a body weight within the normal body mass index range, were free of acute or chronic somatic illnesses, and had no psychiatric comorbidities. Thanks to this design, a highly selective and methodologically homogeneous sample was ensured, enhancing the validity of the findings and allowing for a more precise examination of the relationship between immune markers and clinical presentation in first-episode psychosis. While the number of participants was relatively small, the rigour of the selection process ensured a high degree of internal validity and reduced the influence of potential confounding variables.

In our study, significantly higher concentrations of IL-1β were recorded in the patients with FEDN compared to the healthy controls, which aligns with numerous previous findings indicating a pronounced pro-inflammatory profile in the early stages of psychosis [4,19,20,22]. Across studies, IL-1β levels are consistently elevated in the FEDN patients relative to the controls; this pattern may reflect underlying inflammatory activity rather than being solely a consequence of chronic illness or therapy.

The elevated IL-1β levels in our study can be interpreted in light of modern hypotheses linking inflammation to dysfunctions in neurotransmitter systems in psychosis. According to a systematic review by de Bartolomeis et al. [23], inflammatory cytokines, including IL-1β, may disrupt glutamate regulation in the central nervous system by activating microglia. This disturbance in glutamate homeostasis contributes to NMDA receptor dysfunction, which is considered one of the key mechanisms in the pathogenesis of schizophrenia and related psychoses.

In our study, significantly higher serum concentrations of IL-2 were observed in the patients with FEDN compared to the healthy controls. This finding is consistent with pro-inflammatory activation in the early phase of psychosis and aligns with hypotheses that immune dysregulation is associated with the disorder. As a key cytokine involved in the proliferation and differentiation of T-lymphocytes, IL-2 plays a central role in the Th1 immune response, and its elevated levels have been associated in several studies with adaptive immune activation and potential neuroinflammation [24,25,26]. The literature supporting these findings includes studies conducted on FEDN psychosis patients, which reported elevated IL-2 concentrations and soluble IL-2 receptors (sIL-2R) as indirect markers of T-cell activation [4,15]. However, findings related to IL-2 have not always been consistent. Larger meta-analyses have not identified statistically significant differences in IL-2 levels between patients and healthy individuals [16,20], often attributed to sample heterogeneity, variation in detection methods (e.g., ELISA vs. multiplex platforms), and inconsistent sampling and analysis protocols [17,18]. In this context, our findings—derived from a strictly selected sample with confounding factors eliminated—may reflect a “purer” immune signal, pointing to subtle changes that may have been obscured in the previous studies. This approach highlights the importance of methodological control in inflammation research in psychosis and emphasises the potential diagnostic and research value of IL-2 as a marker of neuroimmune activation in the initial stages of the illness [16,20,27].

In our study, IL-6 concentrations in the patients with FEDN psychosis were not significantly higher compared to the healthy control group. This finding is particularly interesting given that numerous prior studies and meta-analyses have suggested IL-6 as a potential biomarker for psychosis [4,16,20,28]. However, the results have been inconsistent—while some studies consistently reported elevated IL-6 levels, others, including the research by Borovcanin et al. [29], did not find significant differences between patients and healthy individuals. Biologically, IL-6 is a pleiotropic cytokine involved in a broad range of immune and neuroinflammatory processes. It is produced by various cells, including microglia and astrocytes, in response to pathogens, tissue damage, and stress [30,31]. However, its activity is context-dependent: IL-6 has both pro-inflammatory and anti-inflammatory functions. While it stimulates CRP production, it can also inhibit dendritic cell recruitment, stimulate IL-1RA and sTNF-R, and promote alternative macrophage activation, all of which contribute to the resolution of inflammation [30,32]. Our finding aligns with the part of the literature that did not observe elevated IL-6 levels in FEDN psychosis patients [29]. This could be explained by the specific characteristics of our sample. Carefully selected participants—non-smokers, without obesity, somatic or metabolic comorbidities—enabled the minimisation of known modulators of peripheral IL-6 levels [20,33]. This likely accounts for the discrepancy with the prior findings that did not control for these factors. It is also possible that non-elevated IL-6 levels reflect a stage in the inflammatory response in which anti-inflammatory mechanisms are already engaged, or that there is a lack of synchronisation between central and peripheral inflammation, considering that IL-6 plays a vital role in the CNS but may not necessarily be detected in peripheral circulation [31]. Finally, IL-6 fluctuations over time further complicate interpretation; Potvin et al. [34] proposed that such changes may have pathogenetic significance, while Ganguli et al. [35] linked IL-6 levels to disease duration.

In our study, IL-10 levels were found to be significantly elevated in the group of patients with FEDN psychosis compared to the healthy control group. IL-10 is known as a key anti-inflammatory cytokine with a central role in modulating inflammation and maintaining immune homeostasis [36]. Its elevation in our sample may reflect the activation of compensatory mechanisms aimed at limiting the effects of previously identified pro-inflammatory cytokines, such as IL-1β and IL-2. IL-10 functions through negative feedback in the immune response, curbing inflammation and protecting tissues from the damage caused by excessive activation of both the innate and adaptive immune systems [37]. It is produced by various cell populations, including regulatory T (Treg) and B cells, as well as Th2 and Th3 lymphocytes, and is known to inhibit the expression of key pro-inflammatory cytokines, such as IFN-γ, IL-2, and TNF-α [20,37,38]. This IL-10 profile provides a fine-tuned regulation of immune activity, balancing inflammatory and anti-inflammatory pathways [34,37]. The finding of elevated IL-10 in our sample is partially at odds with some studies that have reported predominantly pro-inflammatory profiles in patients with first-episode schizophrenia [20], as well as research identifying reduced IL-10 levels in patients during acute relapses [16,20]. However, our results align with the studies suggesting mixed inflammatory patterns in first episode psychosis patients, involving simultaneous increases in both pro- and anti-inflammatory cytokines [39,40,41].

Existing meta-analyses have suggested that patients with psychosis may exhibit an imbalance between pro-inflammatory cytokines (e.g., IL-1β, IL-6) and anti-inflammatory cytokines (e.g., IL-10), which could underlie immune dysregulation in the early stages of the illness [42,43]. Additionally, studies have shown that elevated prenatal levels of IL-10 and other Th2/anti-inflammatory cytokines in maternal blood may have a protective effect on offspring, reducing the risk of developing schizophrenia [44,45]. A meta-analysis by Zhang et al. [45] specifically highlighted the importance of cytokine imbalance during early pregnancy as a potential risk factor for disease development. It is also important to note that IL-10 and its receptors are synthesised within the central nervous system, including by microglia and astrocytes, where they act as modulators of local inflammatory responses and contribute to the maintenance of homeostasis [46,47,48]. This further complicates the interpretation of peripheral IL-10 levels, as discrepancies may exist between central and peripheral expression at different stages of the illness.

In our study, a significant positive correlation was recorded between elevated levels of IL-1β and the severity of negative symptomatology, as well as general psychopathology scores on the PANSS scale. These findings are consistent with the previous studies that have identified IL-1β as a key pro-inflammatory cytokine associated with more pronounced negative symptoms and overall illness severity [19,20,49]. These results further support observations from the earlier research. For example, a study that did not find differences in IL-1β levels between the patients and the healthy controls still reported positive correlations between IL-1β and the total PANSS scores, including positive, negative, and general psychopathology symptoms within the patient group [50]. Similarly, a meta-analysis by Momtazmanesh et al. [19] demonstrated that IL-1β is associated with higher total PANSS scores, as well as more severe positive symptoms and general psychopathology. Additionally, in our study, a negative correlation was observed between IL-1β levels and the items of grandiosity and hostility on the positive subscale of the PANSS. This finding suggests that higher levels of IL-1β may be associated with lower expression of certain positive symptoms in patients with FEDN psychosis. To the best of our knowledge, the existing literature has not yet described a direct association between IL-1β and these specific dimensions of the clinical picture, making our result a potentially novel contribution to the understanding of inflammation’s role in symptom modulation.

In our study, despite significantly elevated concentrations of IL-2 in the FEDN psychosis patient group compared to the healthy controls, no significant association was found between this cytokine and the overall PANSS score, nor with the individual dimensions of positive, negative, or general psychopathological symptoms. The same applies to IL-6. These findings contrast with numerous other studies that have reported links between these cytokines and psychotic symptoms [51,52,53,54].

In our sample, the absence of a correlation between IL-2 and psychotic symptomatology may suggest that elevated IL-2 levels reflect a general inflammatory response not necessarily directly associated with the severity of the clinical picture in the early stage of illness. As for IL-6, the lack of association with the symptoms in our sample is likely because this cytokine was not elevated compared to the controls, confirming that its effect on the clinical presentation is probably tied to the conditions characterised by more pronounced pro-inflammatory activation.

Within our research, a significant positive correlation was recorded between the concentration of IL-10 and the general psychopathology scale on the PANSS, as well as with the individual symptom of volitional impairment. These findings challenge the simplified interpretation of IL-10 solely as a protective immunomodulator [55], which is a common narrative in the earlier studies that linked lower concentrations of IL-10 with more severe negative symptomatology [56,57]. At the same time, several authors have pointed to a pronounced heterogeneity of findings when it comes to IL-10, which can be explained by differences in the samples, methodology, disease stage, and the treatment status of the participants [19].

In our sample, IL-10 was positively associated with psychopathology and volitional impairment; one possible interpretation is that elevated IL-10 reflects cytokine dysregulation, although this remains speculative. This interpretation is consistent with the studies indicating simultaneously elevated pro- and anti-inflammatory cytokines in patients with first episode psychosis [58]. Elevated IL-10 levels might represent a compensatory response to pro-inflammatory activation, especially alongside increased IL-1β and IL-2, though this cannot be confirmed with the cross-sectional data.

Experimental studies have shown that IL-10 plays an important role in regulating motivation and affect, independently of its anti-inflammatory function. In IL-10 knockout mice, symptoms of depressive behaviour and reduced motivation have been observed, while enhanced IL-10 signalling in the amygdala has been associated with decreased anxiety and the regulation of GABAergic neurotransmission. The experimental evidence indicates that IL-10 can influence emotional and motivational processes; this might partly explain our observed association with volitional impairment, but the mechanistic link remains hypothetical [56,57,58]. Differences between peripheral and central IL-10 signalling have been proposed as a factor in varying clinical presentations of psychosis; our observations may be consistent with this hypothesis, but longitudinal data would be needed to explore it. The correlations between cytokine levels and symptom dimensions were exploratory and not adjusted for multiple comparisons. Consequently, these findings should be interpreted with caution and validated in larger samples. Similarly, group comparisons of cytokine levels were not corrected for multiple testing, which also warrants a cautious interpretation of the results.

The results of the ROC analysis in our study showed that IL-2, IL-1β, and IL-10 had significant discriminatory ability in distinguishing the patients with FEDN psychosis from the healthy controls, while IL-6 did not demonstrate a statistically significant value. The relatively small sample size limits the interpretability of the ROC analysis and should be considered when evaluating the diagnostic utility of the examined biomarkers.

IL-2 showed the highest classification accuracy, indicating its strong potential as a biomarker of acute psychosis. This finding aligns with the results of a meta-analysis by Capuzzi and colleagues, which demonstrated that IL-2 levels significantly decrease following antipsychotic treatment. This positions IL-2 as a state biomarker—i.e., a marker that reflects the current state of the illness and is potentially sensitive to the therapeutic response and phase of the disorder [17]. IL-1β has also demonstrated good discriminatory ability, which can likewise be interpreted in light of its identification as a potential state marker in multiple studies. According to meta-analyses, its concentrations also decrease following treatment, particularly in the early stages of the illness, indicating its association with acute inflammatory activation [17,20]. This perspective is supported by the findings of a recent meta-analysis, which showed that IL-10 levels in patients with FEDN psychosis significantly decrease following antipsychotic treatment [59], suggesting a potential role for IL-10 as a state marker of psychotic episodes—that is, an indicator of an active inflammatory process. At the same time, the authors emphasise that the decrease in IL-10 may also be mediated by the metabolic side effects of antipsychotics, given that obesity and metabolic syndrome are associated with a reduction in anti-inflammatory cytokines, including IL-10. It is also noted that elevated levels of both pro- and anti-inflammatory cytokines are present in the FEDN psychosis phase compared to high-risk groups for psychosis, which may indicate a reactive mobilisation of the immune system during the transition to full psychotic decompensation. By contrast, IL-6 did not show significant discriminatory ability, although several previous meta-analyses have indicated its association with psychosis and sensitivity to treatment [17,20]. The absence of a significant finding in our sample may be due to the specific population, sample size, or the temporal dynamics of the cytokine response. It is also possible that IL-6 shows stronger expression in the later stages of the disease or in patients who have already received treatment.

Taken together, these findings indicate the differential potential of specific interleukins as biomarkers for the early detection of psychosis, with IL-2 and IL-1β most consistently meeting the criteria for state markers, while IL-10 may reflect a more complex immune response involving both proactive and reactive mechanisms during the acute phase of the illness.

## 5. Conclusions

This study further illuminates the immunological profile of patients experiencing a first episode of psychosis, showing that IL-1β, IL-2, and IL-10 are not only significantly altered compared to the healthy controls, but are also associated with specific dimensions of the clinical picture. IL-1β showed a positive correlation with the negative and general scales of the PANSS, as well as a negative association with certain symptoms from the positive subscale, such as grandiosity and hostility. IL-10 was associated with general psychopathology and volitional impairment, while IL-2, although lacking a clear clinical correlation, demonstrated the strongest discriminatory value among the interleukins examined. Although the sample size and the absence of longitudinal follow-up represent methodological limitations, it is also important to emphasise that the analysis focused exclusively on peripheral biomarkers, without the possibility of insight into central inflammatory processes, which leaves open the question of their interrelation and clinical relevance. Another limitation is the difference in marital and employment status between the groups, which may reflect the varying levels of chronic psychosocial stress known to affect inflammation. These factors were not included as covariates due to sample size constraints, but future analyses within the broader study will address their potential confounding effects. According to the available data, specific associations, such as the negative correlation of IL-1β with grandiosity and hostility, as well as the link between IL-10 and the symptom of volitional impairment, have not been consistently reported in the literature so far, which makes these findings a potential new contribution to the understanding of the immunological mechanisms in psychosis. The findings of this study provide preliminary insights into immune dysfunction in the early phase of psychosis and suggest that certain cytokines, particularly IL-2 and IL-1β, may have potential diagnostic relevance. Given the exploratory nature of the symptom-level associations, these observations should be interpreted with caution and require further validation in larger and longitudinal studies. Future research that integrates peripheral and central immune profiles, tracks changes over time, and evaluates the treatment response could contribute to the development of more precise, immune-based strategies for early detection and the personalised treatment of psychotic disorders.

## Figures and Tables

**Table 1:** Demographic data and clinical measures.

Parameter	Healthy Controls	FEDN
Sex n (%)		
Men	14 (63.6)	23 (60.5)
Women	8 (36.4)	15 (39.5)
Age (mean ± SD)	29.91 ± 6.46	27.63 ± 6.84
PANSS (mean ± SD)	NA	
Positive symptoms		29.63 ± 4.77
Negative symptoms		23.45 ± 6.34
General psychopathology		53.47 ± 6.96
Total score		106.55 ± 14.47

PANSS: The Positive and Negative Syndrome Scale; FEDN: First episode drug-naive; SD: standard deviation; NA: not applicable.

**Table 2:** Comparison of Serum Interleukin Concentrations (IL-1β, IL-2, IL-6, and IL-10) Between Patients with Drug-Naive First-Episode Psychosis and Healthy Controls.

Interleukin	Healthy Controls (pg/mL; Mean ± SD)	FEDN (pg/mL; Mean ± SD)	*p*
IL-1β	37.06 ± 27.61	130.14 ± 93.22	0.001
IL-2	42.15 ± 55.59	395.7 ± 380.29	<0.001
IL-6	5.01 ± 4.28	9.13 ± 12.90	0.698
IL-10	24.20 ± 33.43	140.65 ± 176.84	0.004

Serum concentrations of interleukins IL-1β, IL-2, IL-6, and IL-10 are presented as mean ± standard deviation (pg/mL) in the patients with drug-naive first-episode (FEDN) psychosis and the healthy controls. Statistically significant elevations were observed for IL-1β, IL-2, and IL-10 in the patient group compared to the controls (*p* < 0.01 for all), while IL-6 levels did not differ significantly between the groups (*p* = 0.698). All comparisons were performed using the Mann–Whitney U test.

**Table 3:** Diagnostic Performance of Interleukin Serum Levels in Differentiating the Patients with First-Episode Psychosis from the Healthy Controls (ROC Analysis).

Interleukin	AUC	95%CI	Cut Off	Sensitivity (%)	Specificity (%)	*p*
IL-1β	0.842	0.659–0.981	99.18	65.0	100.0	<0.001
IL-2	0.917	0.759–1.000	77.02	95.0	83.3	<0.001
IL-6	0.396	0.187–0.625	13.56	25.0	100.0	0.451
IL-10	0.817	0.565–1.000	44.62	70.0	83.3	<0.001

A receiver operating characteristic (ROC) analysis was performed to evaluate the ability of serum IL-1β, IL-2, IL-6, and IL-10 to discriminate the FEDN group from the healthy controls. Reported values include the optimal cut-off points determined by Youden’s index, area under the curve (AUC) with 95% confidence intervals (CI) obtained via 2000-sample bootstrap resampling, sensitivity, specificity, and *p*-values for the null hypothesis AUC = 0.5.

**Table 4:** Associations Between Serum Interleukin Levels and PANSS Scores in Drug-Naive Patients with First-Episode Psychosis.

PANSS	IL-1β	IL-2	IL-6	IL-10
	ρ/*p*			
Total score	0.146/0.382	0.070/0.688	0.79/0.719	0.225/0.174
Positive symptoms	0.053/0.735	0.046/0.785	0.065/0.733	−0.006/0.970
P1	−0.074/0.660	−0.123/0.483	−0.276/0.202	−0.042/0.804
P2	−0.025/0.880	−0.041/0.815	−0.081/0.715	0.058/0.727
P3	−0.022/0.893	−0.060/0.732	−0.064/0.773	0.078/0.643
P4	−0.082/0.624	−0.087/0.621	−0.122/0.581	−0.087/0.603
P5	**−0.371/0.022**	−0.164/0.348	0.094/0.669	−0.309/0.059
P6	0.031/0.853	0.029/0.867	0.170/0.438	0.026/0.876
P7	**−0.342/0.036**	−0.107/0.540	0.179/0.415	−0.313/0.056
Negative symptoms	**0.373/0.013**	0.218/0.189	0.068/0.722	0.275/0.078
N1	0.222/0.181	0.058/0.741	−0.103/0.642	0.126/0.450
N2	0.065/0.698	0.023/0.895	0.149/0.498	0.204/0.219
N3	0.243/0.142	0.156/0.372	−0.017/0.940	0.302/0.065
N4	0.167/0.317	−0.020/0.909	−0.064/0.770	−0.076/0.650
N5	0.097/0.562	0.083/0.637	−0.112/0.612	0.168/0.314
N6	0.107/0.521	0.059/0.737	0.271/0.211	0.183/0.273
N7	0.104/0.534	−0.146/0.402	−0.078/0.725	0.066/0.696
General psychopathology	**0.375/0.012**	0.265/0.108	0.903/0.626	**0.364/0.018**
G1	0.202/0.224	0.073/0.676	−0.246/0.259	0.159/0.342
G2	0.152/0.362	0.161/0.354	0.175/0.435	0.191/0.252
G3	0.219/0.186	0.071/0.686	−0.306/0.156	0.110/0.512
G4	−0.172/0.302	0.051/0.770	0.403/0.056	0.043/0.797
G5	0.092/0.584	0.084/0.632	0.122/0.579	0.198/0.234
G6	0.209/0.208	0.135/0.438	−0.144/0.511	0.125/0.455
G7	0.235/0.155	−0.035/0.840	0.026/0.905	0.195/0.242
G8	−0.034/0.841	0.017/0.922	0.007/0.795	−0.067/0.690
G9	−0.089/0.596	−0.031/0.859	−0.024/0.914	−0.004/0.983
G10	0.133/0.427	0.105/0.548	0.225/0.302	0.193/0.245
G11	−0.026/0.875	−0.105/0.548	−0.099/0.654	0.001/0.997
G12	−0.002/0.541	0.023/0.897	−0.047/0.832	0.021/0.899
G13	0.159/0.341	0.097/0.580	0.381/0.073	**0.342/0.036**
G14	−0.149/0.370	−0.062/0.726	−0.017/0.938	−0.008/0.961
G15	0.103/0.540	−0.045/0.799	0.232/0.287	0.227/0.170
G16	0.190/0.252	0.115/0.512	0.247/0.256	0.273/0.098

Spearman’s correlation coefficients (ρ) and *p*-values for associations between serum levels of IL-1β, IL-2, IL-6, and IL-10, and the PANSS total score, subscales (positive, negative, general psychopathology), and individual symptom items in drug-naive patients with first-episode psychosis (FEDN). IL-1β showed significant positive correlations with negative and general psychopathology dimensions, as well as negative correlations with specific positive symptoms (grandiosity and hostility). IL-10 was positively associated with the total PANSS score and the item “disturbance of volition”. IL-2 and IL-6 showed limited or no significant associations with clinical symptoms. Bold values indicate statistically significant results (*p* < 0.05).

## Data Availability

The data presented in this study are available upon request from the corresponding author. The data are not publicly available due to ethical restrictions and concerns related to participant confidentiality, as the dataset includes sensitive clinical and biological variables obtained from individuals with psychiatric illness.

## Abbreviations

The following abbreviations are used in this manuscript:

PANSS	Positive and Negative Syndrome Scale
FEDN	First-Episode Drug-Naive
IL	Interleukin

## References

[1] Al-Diwani A.A.J., Pollak T.A., Irani S.R., Lennox B.R. (2017). Psychosis: An autoimmune disease?. Immunology.

[2] Dunleavy C., Elsworthy R.J., Upthegrove R., Wood S.J., Aldred S. (2022). Inflammation in first-episode psychosis: The contribution of inflammatory biomarkers to the emergence of negative symptoms, a systematic review and meta-analysis. Acta Psychiatr. Scand..

[3] Khandaker G.M., Cousins L., Deakin J., Lennox B.R., Yolken R., Jones P.B. (2015). Inflammation and immunity in schizophrenia: Implications for pathophysiology and treatment. Lancet Psychiatry.

[4] Upthegrove R., Manzanares-Teson N., Barnes N.M. (2014). Cytokine function in medication-naive first episode psychosis: A systematic review and meta-analysis. Schizophr. Res..

[5] Foley É.M., Khandaker G.M. (2023). Cytokines in psychosis: From mechanism towards treatment and prediction. Lancet Psychiatry.

[6] Sekar A., Bialas A.R., De Rivera H., Davis A., Hammond T.R., Kamitaki N., Tooley K., Presumey J., Baum M., Van Doren V. (2016). Schizophrenia risk from complex variation of complement component 4. Nature.

[7] Miller B.J., Goldsmith D.R. (2020). Evaluating the Hypothesis That Schizophrenia Is an Inflammatory Disorder. Focus.

[8] Feigenson K.A., Kusnecov A.W., Silverstein S.M. (2014). Inflammation and the two-hit hypothesis of schizophrenia. Neurosci. Biobehav. Rev..

[9] Read J., Van Os J., Morrison A.P., Ross C.A. (2005). Childhood trauma, psychosis and schizophrenia: A literature review with theoretical and clinical implications. Acta Psychiatr. Scand..

[10] Liu C., Chu D., Kalantar-Zadeh K., George J., Young H.A., Liu G. (2021). Cytokines: From Clinical Significance to Quantification. Adv. Sci..

[11] Pollak T.A., Drndarski S., Stone J.M., David A.S., McGuire P., Abbott N.J. (2018). The blood–brain barrier in psychosis. Lancet Psychiatry.

[12] Dinarello C.A. (2011). A clinical perspective of IL-1β as the gatekeeper of inflammation. Eur. J. Immunol..

[13] Shen Y., Zhu L.J., Liu S.S., Zhou S.Y., Luo J.H. (2006). Interleukin-2 inhibits NMDA receptor-mediated currents directly and may differentially affect subtypes. Biochem. Biophys. Res. Commun..

[14] Pavăl D., Gherghel-Pavăl N., Căpățînă O.O., Stan A., Micluția I.V., Giné-Servén E. (2023). The Importance of Cerebrospinal Fluid Investigation in First-episode Psychosis. Yale J. Biol. Med..

[15] Müller N., Weidinger E., Leitner B., Schwarz M.J. (2015). The role of inflammation in schizophrenia. Front. Neurosci..

[16] Goldsmith D.R., Rapaport M.H., Miller B.J. (2016). A meta-analysis of blood cytokine network alterations in psychiatric patients: Comparisons between schizophrenia, bipolar disorder and depression. Mol. Psychiatry.

[17] Capuzzi E., Bartoli F., Crocamo C., Clerici M., Carrà G. (2017). Acute variations of cytokine levels after antipsychotic treatment in drug-naïve subjects with a first-episode psychosis: A meta-analysis. Neurosci. Biobehav. Rev..

[18] Romeo B., Brunet-Lecomte M., Martelli C., Benyamina A. (2018). Kinetics of Cytokine Levels during Antipsychotic Treatment in Schizophrenia: A Meta-Analysis. Int. J. Neuropsychopharmacol..

[19] Momtazmanesh S., Zare-Shahabadi A., Rezaei N. (2019). Cytokine Alterations in Schizophrenia: An Updated Review. Front. Psychiatry.

[20] Miller B.J., Buckley P., Seabolt W., Mellor A., Kirkpatrick B. (2011). Meta-Analysis of Cytokine Alterations in Schizophrenia: Clinical Status and Antipsychotic Effects. Biol. Psychiatry.

[21] Pillinger T., D’Ambrosio E., McCutcheon R., Howes O.D. (2018). Is psychosis a multisystem disorder? A meta-review of central nervous system, immune, cardiometabolic, and endocrine alterations in first-episode psychosis and perspective on potential models. Mol. Psychiatry.

[22] Di Nicola M., Cattaneo A., Hepgul N., Di Forti M., Aitchison K.J., Janiri L., Murray R.M., Dazzan P., Pariante C.M., Mondelli V. (2013). Serum and gene expression profile of cytokines in first-episode psychosis. Brain Behav. Immun..

[23] de Bartolomeis A., Barone A., Vellucci L., Mazza B., Austin M.C., Iasevoli F., Ciccarelli M. (2022). Linking Inflammation, Aberrant Glutamate-Dopamine Interaction, and Post-synaptic Changes: Translational Relevance for Schizophrenia and Antipsychotic Treatment: A Systematic Review. Mol. Neurobiol..

[24] Boerrigter D., Weickert T.W., Lenroot R., O’Donnell M., Galletly C., Liu D., Burgess M., Cadiz R., Jacomb I., Catts V.S. (2017). Using blood cytokine measures to define high inflammatory biotype of schizophrenia and schizoaffective disorder. J. Neuroinflamm..

[25] Carril Pardo C., Oyarce Merino K., Vera-Montecinos A. (2025). Neuroinflammatory Loop in Schizophrenia, Is There a Relationship with Symptoms or Cognition Decline?. Int. J. Mol. Sci..

[26] Hanisch U.K., Quirion R. (1995). Interleukin-2 as a neuroregulatory cytokine. Brain Res. Rev..

[27] Misiak B., Bartoli F., Carrà G., Stańczykiewicz B., Gładka A., Frydecka D., Samochowiec J., Jarosz K., Hadryś T., Miller B.J. (2021). Immune-inflammatory markers and psychosis risk: A systematic review and meta-analysis. Psychoneuroendocrinology.

[28] Stojanovic A., Martorell L., Montalvo I., Ortega L., Monseny R., Vilella E., Labad J. (2014). Increased serum interleukin-6 levels in early stages of psychosis: Associations with at-risk mental states and the severity of psychotic symptoms. Psychoneuroendocrinology.

[29] Borovcanin M., Jovanovic I., Radosavljevic G., Djukic Dejanovic S., Bankovic D., Arsenijevic N., Lukic M.L. (2012). Elevated serum level of type-2 cytokine and low IL-17 in first episode psychosis and schizophrenia in relapse. J. Psychiatr. Res..

[30] Tanaka T., Narazaki M., Kishimoto T. (2014). Il-6 in inflammation, Immunity, And disease. Cold Spring Harb. Perspect. Biol..

[31] Borovcanin M.M., Jovanovic I., Radosavljevic G., Pantic J., Janicijevic S.M., Arsenijevic N., Lukic M.L., Borovcanin M.M., Jovanovic I., Radosavljevic G. (2017). Interleukin-6 in schizophrenia-Is there a therapeutic relevance?. Front. Psychiatry.

[32] Scheller J., Chalaris A., Schmidt-Arras D., Rose-John S. (2011). The pro- and anti-inflammatory properties of the cytokine interleukin-6. Biochim. Biophys. Acta Mol. Cell Res..

[33] Khandaker G.M., Zammit S., Burgess S., Lewis G., Jones P.B. (2018). Association between a functional interleukin 6 receptor genetic variant and risk of depression and psychosis in a population-based birth cohort. Brain Behav. Immun..

[34] Potvin S., Stip E., Sepehry A.A., Gendron A., Bah R., Kouassi E. (2008). Inflammatory Cytokine Alterations in Schizophrenia: A Systematic Quantitative Review. Biol. Psychiatry.

[35] Ganguli R., Yang Z., Shurin G., Chengappa K.N.R., Brar J.S., Gubbi A.V., Rabin B.S. (1994). Serum interleukin-6 concentration in schizophrenia: Elevation associated with duration of illness. Psychiatry Res..

[36] Carlini V., Noonan D.M., Abdalalem E., Goletti D., Sansone C., Calabrone L., Albini A. (2023). The multifaceted nature of IL-10: Regulation, role in immunological homeostasis and its relevance to cancer, COVID-19 and post-COVID conditions. Front. Immunol..

[37] Moore K.W., De Waal Malefyt R., Coffman R.L., O’Garra A. (2001). Interleukin-10 and the interleukin-10 receptor. Annu. Rev. Immunol..

[38] Severance E.G., Gressitt K.L., Stallings C.R., Origoni A.E., Khushalani S., Leweke F.M., Dickerson F.B., Yolken R.H. (2013). Discordant patterns of bacterial translocation markers and implications for innate immune imbalances in schizophrenia. Schizophr. Res..

[39] Kunz M., Ceresér K.M., Goi P.D., Fries G.R., Teixeira A.L., Fernandes B.S., Belmonte-De-Abreu P.S., Kauer-Sant’Anna M., Kapczinski F., Gama C.S. (2011). Serum levels of IL-6, IL-10 and TNF-α in patients with bipolar disorder and schizophrenia: Differences in pro- and anti-inflammatory balance. Braz. J. Psychiatry.

[40] Schwarz E., Guest P.C., Rahmoune H., Harris L.W., Wang L., Leweke F.M., Rothermundt M., Bogerts B., Koethe D., Kranaster L. (2012). Identification of a biological signature for schizophrenia in serum. Mol. Psychiatry.

[41] Maes M., Bocchio Chiavetto L., Bignotti S., Battisa Tura G.J., Pioli R., Boin F., Kenis G., Bosmans E., De Jongh R., Altamura C.A. (2002). Increased serum interleukin-8 and interleukin-10 in schizophrenic patients resistant to treatment with neuroleptics and the stimulatory effects of clozapine on serum leukemia inhibitory factor receptor. Schizophr. Res..

[42] Trovão N., Prata J., Vondoellinger O., Santos S., Barbosa M., Coelho R. (2019). Peripheral Biomarkers for First-Episode Psychosis—Opportunities from the Neuroinflammatory Hypothesis of Schizophrenia. Psychiatry Investig..

[43] Barron H., Hafizi S., Andreazza A.C., Mizrahi R. (2017). Neuroinflammation and Oxidative Stress in Psychosis and Psychosis Risk. Int. J. Mol. Sci..

[44] Allswede D.M., Buka S.L., Yolken R.H., Torrey E.F., Cannon T.D. (2016). Elevated maternal cytokine levels at birth and risk for psychosis in adult offspring. Schizophr. Res..

[45] Zhang J., Luo W., Huang P., Peng L., Huang Q. (2018). Maternal C-reactive protein and cytokine levels during pregnancy and the risk of selected neuropsychiatric disorders in offspring: A systematic review and meta-analysis. J. Psychiatr. Res..

[46] Sawada M., Suzumura A., Hosoya H., Marunouchi T., Nagatsu T. (1999). Interleukin-10 inhibits both production of cytokines and expression of cytokine receptors in microglia. J. Neurochem..

[47] Strle K., Zhou J.H., Shen W.H., Broussard S.R., Johnson R.W., Freund G.G., Dantzer R., Kelley K.W. (2001). Interleukin-10 in the brain. Crit. Rev. Immunol..

[48] Ledeboer A., Brevé J.J.P., Wierinckx A., Van Der Jagt S., Bristow A.F., Leysen J.E., Tilders F.J.H., Van Dam A. (2002). Expression and regulation of interleukin-10 and interleukin-10 receptor in rat astroglial and microglial cells. Eur. J. Neurosci..

[49] Dai N., Jie H., Duan Y., Xiong P., Xu X., Chen P., Kang M., Li M., Li T., Huang Z. (2020). Different serum protein factor levels in first-episode drug-naive patients with schizophrenia characterized by positive and negative symptoms. Psychiatry Clin. Neurosci..

[50] Mednova I.A., Boiko A.S., Kornetova E.G., Semke A.V., Bokhan N.A., Ivanova S.A. (2022). Cytokines as Potential Biomarkers of Clinical Characteristics of Schizophrenia. Life.

[51] Asevedo E., Rizzo L.B., Gadelha A., Mansur R.B., Ota V.K., Berberian A.A., Scarpato B.S., Teixeira A.L., Bressan R.A., Brietzke E. (2014). Peripheral interleukin-2 level is associated with negative symptoms and cognitive performance in schizophrenia. Physiol. Behav..

[52] Zhilyaeva T., Rukavishnikov G., Manakova E., Mazo G. (2023). Serum Interleukin-6 in Schizophrenia: Associations with Clinical and Sociodemographic Characteristics. Consort. Psychiatr..

[53] Chase K.A., Cone J.J., Rosen C., Sharma R.P. (2016). The value of interleukin 6 as a peripheral diagnostic marker in schizophrenia. BMC Psychiatry.

[54] Frydecka D., Misiak B., Pawlak-Adamska E., Karabon L., Tomkiewicz A., Sedlaczek P., Kiejna A., Beszłej J.A. (2015). Interleukin-6: The missing element of the neurocognitive deterioration in schizophrenia? The focus on genetic underpinnings, cognitive impairment and clinical manifestation. Eur. Arch. Psychiatry Clin. Neurosci..

[55] Couper K.N., Blount D.G., Riley E.M. (2008). IL-10: The Master Regulator of Immunity to Infection. J. Immunol..

[56] Şimşek Ş., Ylldlrlm V., Çim A., Kaya S. (2016). Serum IL-4 and IL-10 Levels Correlate with the Symptoms of the Drug-Naive Adolescents with First Episode, Early Onset Schizophrenia. J. Child Adolesc. Psychopharmacol..

[57] Xiu M.H., Yang G.G., Tan Y.L., Chen D.C., Tan S.P., Wang Z.R., De Yang F., Okusaga O., Soares J.C., Zhang X.Y. (2014). Decreased interleukin-10 serum levels in first-episode drug-naïve schizophrenia: Relationship to psychopathology. Schizophr. Res..

[58] García-Bueno B., Bioque M., Mac-Dowell K.S., Barcones M.F., Martínez-Cengotitabengoa M., Pina-Camacho L., Rodríguez-Jiménez R., Sáiz P.A., Castro C., Lafuente A. (2014). Pro-/Anti-inflammatory Dysregulation in Patients with First Episode of Psychosis: Toward an Integrative Inflammatory Hypothesis of Schizophrenia. Schizophr. Bull..

[59] Marcinowicz P., Więdłocha M., Zborowska N., Dębowska W., Podwalski P., Misiak B., Tyburski E., Szulc A. (2021). A Meta-Analysis of the Influence of Antipsychotics on Cytokines Levels in First Episode Psychosis. J. Clin. Med..

